# Mobile Applications for Diabetics: A Systematic Review and Expert-Based Usability Evaluation Considering the Special Requirements of Diabetes Patients Age 50 Years or Older

**DOI:** 10.2196/jmir.2968

**Published:** 2014-04-09

**Authors:** Madlen Arnhold, Mandy Quade, Wilhelm Kirch

**Affiliations:** ^1^Research Association Public Health Saxony and Saxony-AnhaltMedizinische Fakultät Carl Gustav CarusTechnische Universität DresdenDresdenGermany

**Keywords:** mobile applications, apps, mobile health, mHealth, diabetes mellitus, market analysis, systematic review, elderly, usability test, expert review

## Abstract

**Background:**

A multitude of mhealth (mobile health) apps have been developed in recent years to support effective self-management of patients with diabetes mellitus type 1 or 2.

**Objective:**

We carried out a systematic review of all currently available diabetes apps for the operating systems iOS and Android. We considered the number of newly released diabetes apps, range of functions, target user groups, languages, acquisition costs, user ratings, available interfaces, and the connection between acquisition costs and user ratings. Additionally, we examined whether the available applications serve the special needs of diabetes patients aged 50 or older by performing an expert-based usability evaluation.

**Methods:**

We identified relevant keywords, comparative categories, and their specifications. Subsequently, we performed the app review based on the information given in the Google Play Store, the Apple App Store, and the apps themselves. In addition, we carried out an expert-based usability evaluation based on a representative 10% sample of diabetes apps.

**Results:**

In total, we analyzed 656 apps finding that 355 (54.1%) offered just one function and 348 (53.0%) provided a documentation function. The dominating app language was English (85.4%, 560/656), patients represented the main user group (96.0%, 630/656), and the analysis of the costs revealed a trend toward free apps (53.7%, 352/656). The median price of paid apps was €1.90. The average user rating was 3.6 stars (maximum 5). Our analyses indicated no clear differences in the user rating between free and paid apps. Only 30 (4.6%) of the 656 available diabetes apps offered an interface to a measurement device. We evaluated 66 apps within the usability evaluation. On average, apps were rated best regarding the criterion “comprehensibility” (4.0 out of 5.0), while showing a lack of “fault tolerance” (2.8 out of 5.0). Of the 66 apps, 48 (72.7%) offered the ability to read the screen content aloud. The number of functions was significantly negative correlated with usability. The presence of documentation and analysis functions reduced the usability score significantly by 0.36 and 0.21 points.

**Conclusions:**

A vast number of diabetes apps already exist, but the majority offer similar functionalities and combine only one to two functions in one app. Patients and physicians alike should be involved in the app development process to a greater extent. We expect that the data transmission of health parameters to physicians will gain more importance in future applications. The usability of diabetes apps for patients aged 50 or older was moderate to good. But this result applied mainly to apps offering a small range of functions. Multifunctional apps performed considerably worse in terms of usability. Moreover, the presence of a documentation or analysis function resulted in significantly lower usability scores. The operability of accessibility features for diabetes apps was quite limited, except for the feature “screen reader”.

## Introduction

Compared to early mobile phones, today’s smartphones and tablet PCs offer a considerably wider range of functionalities. Mobile applications (apps) are increasingly used in managing various tasks in daily life. Currently, more than 900,000 apps are available in the Apple App Store (operating system: iOS, developer: Apple) and more than 700,000 apps in the Google Play Store (operating system: Android, developer: Google) [1]. The number of health-related apps increased to 31,000 in 2013 [2].

Within the health care sector, apps are supporting the management of illnesses, thereby promoting health awareness and well-being [3-5]. Specifically, a multitude of apps have been developed to assist patients in the management of diabetes mellitus type 1 or 2 [3,6]. For example, a topic-specific search in the Google Play Store resulted in more than 1000 hits. The ability, however, to sort the results according to individual needs is lacking. One reason for the large number of diabetes apps is the high and steadily increasing diabetes prevalence, especially among people older than 50 years [7-9]. In 2012, 371 million people between the ages of 20 and 79 suffered from diabetes worldwide and this number is estimated to increase to 552 million people by 2030 [10]. The high self-therapy potential certainly has a major influence on the high number of currently available apps.

We carried out a systematic review of all currently available diabetes apps for the operating systems iOS and Android, between February 2013 and April 2013. Our review aimed to provide an overview of the number of newly released apps, range of functions, target user groups, languages, acquisition costs, popularity/user ratings, the ability to connect to measurement devices, and the connection between acquisition costs and user ratings.

Diabetes prevalence increases with age. Thus, the elderly are a large target group that could benefit from diabetes apps. However, several studies have shown a lack of acceptance and a subpar use of innovative mobile technologies among this age group [11-15]. As one possible reason, Holzinger et al [11] and Mallenius et al [13] have pointed out the insufficient consideration of usability requirements of the elderly. Their experiences in handling mobile devices and apps are frequently limited. Inhibition thresholds and entry barriers are therefore particularly pronounced among this age group. In addition, cognitive and physical skills are declining with age [11] and result in needs that are considerably different from those of young users. Hence, this age group would benefit from apps that consider their specific usability requirements.

In order to better assess and quantify usability for the elderly, we carried out an expert-based usability evaluation based on a representative 10% sample of diabetes apps available as of April 2013. Therewith, we examined to what extent existing diabetes applications serve the usability requirements of diabetes patients aged 50 or older.

Until now, just a few reviews of diabetes apps had been conducted [3,5,6,16]. They differ from the review presented here in several ways: they considered a broader range of health care applications, they were restricted to one operating system, they reviewed solely the offered functionalities, or they were done more than one year ago. There is an absence of usability evaluation for diabetes apps [16,17]. Especially, formative usability evaluations of health apps are rare. To our knowledge, just one article has been published that links a diabetes app review with a formative usability evaluation [16], but their evaluation is limited to Android apps and gives no special consideration to the requirements of elderly diabetes patients as we do.

## Methods

### Systematic Review

#### Search and Screening Strategy

Our review focused on the leading operating systems for mobile devices, iOS and Android. The analysis was carried out using the Apple App Store for iOS apps and the Google Play Store for Android apps. We focused exclusively on diabetes apps available in English and German.

As a first step, we identified keywords to ensure that every relevant diabetes app was detected. Therefore, we chose the following German and English keywords, directly related to diabetes mellitus: Diabetes, Blood Sugar/Blutzucker, Glucose/Glukose. Every hit was reviewed in terms of its relevance and explicit link to diabetes mellitus. This pre-selection was necessary due to the growing number of misleading descriptions (spam techniques) for apps, caused partly by non-existent or low admission requirements for novel apps. In the Google Play Store, no admission requirements currently exist for newly developed apps, whereas iOS apps are first internally reviewed by an app review board. All apps with an explicit link to diabetes mellitus were included in the analysis. The basis for the systematic and comparative market analysis was defined by categories and respective subcategories/specifications outlined in Table 1.

We considered all the available information given by both the stores and the apps and collected the information for all categories and subcategories/specifications. In some cases, the structure of the app stores and the provided information differed strongly from one another, so we applied different approaches for the analysis of iOS and Android apps.

**Table 1:** Categories and respective subcategories/specifications extracted from diabetes apps.

Category	Subcategory/specifications
**General information**	
	App name
	App language
	Date of release/date of latest update (the acquisition of the release date was only possible for iOS apps; for Android apps, only the date of the latest update could be recorded)
	Availability of a desktop application
**Operating system**	
	App exclusively for the iOS operating system
	App exclusively for the Android operating system
	App for both operating systems available
**Developer information**	
	Name of the developer
**Acquisition costs**	
	Freeware
	Exact price
	Availability as “lite” version (paid apps sometimes offer free or cheaper lite versions with limited functionality)
**Popularity/user ratings**	
	Number of downloads/installations
	User rating
	Number of user ratings
**Range of functions (multiple selection possible)**	
	Documentation function
	Information function
	Data forwarding/communication function
	Analysis function
	Recipe suggestions
	Reminder function/timer
	Advisory function/therapy support
**Target user groups**	
	Patients
	Physicians/qualified health personnel
	Both user groups
**Interfaces**	
	Availability of an interface/connectivity to an external sensor(s)/device

#### Search and Screening Strategy for iOS Apps

The analysis of iOS apps was conducted using the information available in the Apple App Store. In contrast to the Google Play Store, the Apple App Store offers several options for filtering the search results by choosing thematic subcategories. The results can additionally be sorted by relevance, popularity, user rating, and date of release. During the survey period, a sorting function was only available for the iPad, so the whole iOS app survey was performed via the iPad.

For the analysis, we chose the subcategories “Health and Fitness” and “Medicine”. Subsequently, the displayed apps were sorted by their date of release. The date of release served as an objective characteristic, which was necessary for a reliable and reproducible acquisition of all diabetes apps. The number of hits given by the Apple App Store corresponded exactly to the number of relevant apps.

We checked every app hit with regard to its availability for iPad and iPhone. Additionally, we verified whether the app was offered exclusively for the operating system iOS or also for Android. The market analysis of diabetes apps for iOS resulted in 390 hits.

#### Search and Screening Strategy for Android Apps

By using the information available in the Google Play Store, the analysis of Android apps was conducted. To date, this app store offers no option to filter the search results for apps according to individual needs. Furthermore, the given “numbers of hits” is not only the number of apps but also the number of detected search terms in the app title and the app description. Thus, the search term “diabetes” led to more than 1000 hits in the Google Play Store. Keeping the limitations in mind, the number of available apps was a considerable overestimation.

In order to ensure a representative analysis despite missing selection criteria, we defined one day (03/06/2013) to record all found apps with title and developer. This definition will enable future app review processes. Additionally, every app was crosschecked for availability of an iOS version. Altogether, we found 380 diabetes apps available for the operating system Android.

### Expert-Based Usability Evaluation

To examine the usability of currently available diabetes applications for the elderly, we performed an expert-based usability evaluation. With this method, usability experts put themselves in the role of potential or current users to examine products in terms of usability. We performed a summative evaluation as we exclusively included apps whose development was already finished [18].

Due to the high number of apps available for review, the usability evaluation was based on a representative 10% sample of existing diabetes apps as of April 2013. The sample was chosen on a random basis. The evaluation was performed by three independent experts, as suggested by Nielsen [19] and Barnum [20]. They were chosen due to their comprehensive experience in handling and testing mobile devices and applications with regard to usability for the elderly and operability of accessibility features. In addition, they had specific expertise in the field of diabetes and diabetes management. They were already involved in the accompanying systematic review and a survey of diabetes patients aged 50 or older and physicians investigating the acceptance factors of diabetes applications. The authors of this article were not involved in the usability evaluation for reasons of independence.

The basis for the usability evaluation was defined by a specially created set of usability criteria considering interaction processes, interface design, and comprehensibility of content (Table 2). Therefore, we reviewed usability guidelines (ISO, DIN) with explicit regard to the requirements of the elderly concerning mobile applications. Additionally, we considered usability requirements that have been proven as relevant in previous studies with this age group (Table 2). This guideline-based approach of usability testing is in accordance with the proposals of Nielsen [19], and Sarodnick and Brau [18]. We divided the selected criteria into main and subcriteria, added a clear description of their specific characteristics and defined respective assessment criteria. The experts rated each subcriterion and the expression of its characteristics by means of a 5-point Likert scale to grade the evaluation or by means of a dichotomous scale [16,19-21]. The main criteria were not evaluated themselves; their scores were calculated from the mean of the respective subcriteria.

To lower barriers for persons with reduced or limited cognitive and physical skills, iOS and Android offer different accessibility features. We tested the operability of three features for each tested app in a separate test run. We have chosen features that are relevant to the elderly and were offered by both operating systems:

- Screen reader—Voice over (iOS)/Talk back (Android): dichotomous scale
- “Larger Type” as an additional measurement for “possibility to flexibly adapt the size of operating elements and displayed images”: dichotomous scale
- “Invert colors” as an additional measurement for “sufficient color contrast”: 5-point Likert scale

According to the methodical approach of Barnum, the evaluators run through typical scenarios of use to conduct their evaluation [20]. They were asked to take the perspective of a diabetes patient aged 50 or older. Each expert tested the main functionalities of the app, listed in the app description (eg, record of blood glucose data and/or medication, plotting graphs, search for information on diabetes mellitus, etc). All Android apps were tested on a Samsung Galaxy Note 10.1. All iOS apps were tested on an iPad 4^th^generation. If apps were offered for multiple platforms by the manufacturer, they were tested on an iPad 4^th^generation.

The chosen method offers a high level of validity and comparability due to its guideline-based approach and closed response categories [22]. Additionally, a user-based test would not have been able to represent the pronounced heterogeneity among the age group 50 or older regarding health status, skills and preferences, experience in technology use, sociodemographics, etc, which is much more pronounced than for younger age groups [13,15,23-25]. At the same time, it would have been rather difficult to find test persons whose characteristics corresponded exactly to the distribution within the basic population.

**Table 2:** Evaluated usability and assessment criteria for diabetes apps for the elderly.

Main criterion/subcriteria		Description of characteristics	Assessment criteria
**Comprehensibility**			
	**Use of understandable semantics**		
		Avoidance of foreign language and technical terms	5-point Likert scale (1=does not apply at all; 5=does fully apply)
		Use of generally intelligible symbols and terms	
		If necessary, provision of additional explanations [14,26,27]	
	**Simple comprehensibility and interpretability of displayed images and depictions**		
		Self-explanatory images and depictions, understandable without further support and explanations [12]	5-point Likert scale (1=does not apply at all; 5= does fully apply)
	**Simple, self-explanatory menu structures**		
		Easily understandable and internally consistent menu structures	5-point Likert scale (1=does not apply at all; 5=does fully apply)
		Avoidance of strong hierarchical menu structures and too many functionalities [11,15,28]	
**Presentation (Image and Text)**			
	**Sufficient color contrast**		
		Clear, distinguishable colors for images and depictions or choice of color-neutral depictions	5-point Likert scale (1=does not apply at all; 5=does fully apply)
		Avoidance of too glaring colors [12,26]	
	**Large size of operating elements**		
		Sufficient size of screen as well as input and output fields [13,27,28]	5-point Likert scale (1=does not apply at all; 5=does fully apply)
	**Ability to adapt the size of operating elements and displayed images**		
		Ability to adapt size of operating elements and displayed images according to individual needs, capabilities, and preferences [14,26]	Dichotomous scale (applicable, not applicable)
**Usability**			
	**Instant and easily understandable feedback**		
		Instant response to entered data, including easily understandable error messages in case of erroneous data input [15]	5-point Likert scale (1=does not apply at all; 5=does fully apply)
	**Intuitive usability**		
		Ability to use the application without prior knowledge	5-point Likert scale (1=does not apply at all; 5=does fully apply)
		Ease of learning	
		Fast achievement of a first feeling of success [15,29]	
	**Simple recognition of click-sensitive areas**		
		Simple distinction between click-sensitive and non-click-sensitive areas, also without prior knowledge of the features of the touchscreen technology [12]	5-point Likert scale (1=does not apply at all; 5=does fully apply)
**General characteristics**			
	**High fault tolerance/efficient fault management**		
		Reducing probability of erroneous data input by limiting choice to meaningful values	5-point Likert scale (1=does not apply at all; 5=does fully apply)
		Efficient proofreading mode and/or helpful user feedback, for example, in case of erroneous data input [27,30]	
	**Password-protected services**		
		Avoidance of registration at online platforms (but partly contrary to data protection regulations) [13]	Dichotomous scale (applicable, not applicable)

## Results

### Systematic Review

#### Search and Screening

In total, we examined 656 apps during the review process. As a result, we created three data sets (Multimedia Appendix 1), which separated the currently available diabetes apps into apps available exclusively for the operating system iOS (276 apps), apps available exclusively for the operating system Android (266 apps), and apps available for both operating systems (114 apps).

#### Annual Development of App Releases

**Figure 1:**
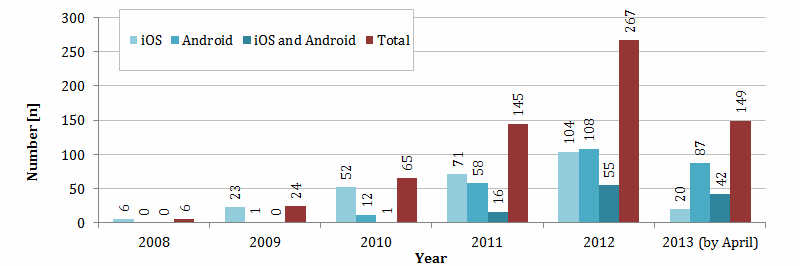
Annual release figures for diabetes apps.

The first diabetes app for iOS (according to Apple App Store as of April 2013) was developed and released on July 17, 2008 (name: Glucose-Charter*,* developer: e-agent). The first Android diabetes app (according to Google Play Store as of April 2013) followed on November 8, 2009 (name: Body Sugar, developer: Adibu). The number of diabetes apps released annually increased during the last five years, from 6 in 2008 to 267 in 2012. In the first four months of 2013, 149 new diabetes apps were released. The number of apps for Android more than doubled each year (Figure 1); however, this was not by publication date (unavailable in Google Play Store) but rather the date of the last update. More than half of the iOS diabetes apps (50.7%, 140/276) were specially designed for use on the iPhone. Only 87/276 (31.5%) were designed for both iPhone and iPad. Due to a lack of information in the Google Play Store, this subdivision into smartphone and tablet PC apps could not be made for Android apps.

#### Operating Language

The majority (85.4%, 560/656) of the examined apps were in English, especially the apps running exclusively on an Android operating system, (90.2%, 240/266). Apps with German as operating language were of relatively low number (14.6%, 96/656) (Table 3). Some apps offered the opportunity to choose between several languages after download.

**Table 3:** Language of available diabetes apps as of April 2013.

		Operating system			
Category	Subcategory	iOS (n=276)	Android (n=266)	iOS and Android (n=114)	Total (n=656)
**Language, n (%)**					
	English	229 (83.0)	240 (90.2)	91 (79.8)	560 (85.4)
	German	47 (17.0)	26 (9.8)	23 (20.2)	96 (14.6)

#### Acquisition Costs

The acquisition costs and the ratio of free to paid apps differed strongly between the two operating systems (Table 4). While most of the iOS apps required payment (62.3%, 172/276), the vast majority of Android and Android/iOS apps were free (63.5%, 169/266 and 69.3%, 79/114). Nonetheless, some of the free apps and the inexpensive apps worked with specially designed test strips or were able to be linked to measurement devices. In these cases, the apps could not have been used without compatible devices.

The analysis of app price distribution revealed that a greater number of free apps were available across all apps (53.7%, 352/656). This appeared to be driven by Android apps where 63.5% (169/266) were free compared with 36.5% (97/266) paid. The reverse trend was observed for iOS where only 37.7% (104/276) were free compared with 62.3% (172/276) paid (Table 4).

**Figure 2:**
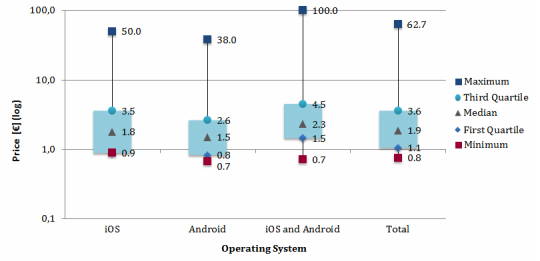
Price distribution of paid diabetes apps available as of April 2013.

The price of paid apps differed strongly between the operating systems (Figure 2). The vast majority (69.7%, 212/304), were in the price range of €0.01 to €3.00. The median price varied between €1.50 and €2.30, depending on the operating system. The apps designed for both operating systems tended to be the apps with the highest price level (Figure 2). The analysis also showed that some costly apps offer free or cheaper “lite” versions with limited functionalities (5.3%, 35/656).

**Table 4:** Price distribution of apps and annual proportions of free apps since 2008.

Category	Subcategory	Operating system			
		iOS (n=276)	Android (n=266)	iOS and Android (n=114)	Total (n=656)
**Price distribution of diabetes apps and “lite” versions, n (%)**					
	Free	104 (37.7)	169 (63.5)	79 (69.3)	352 (53.7)
	Paid	172 (62.3)	97 (36.5)	35 (30.7)	304 (46.3)
	Paid/Lite version available	18 (6.5)	11 (4.1)	6 (5.3)	35 (5.3)
**Development share of free diabetes apps since 2008, n (%)**					
	2013 (by April)	6/20 (30.0)	60/87 (69.0)	33/42 (78.6)	99/149 (66.4)
	2012	58/104 (55.8)	79/108 (73.7)	40/55 (72.7)	177/267 (66.3)
	2011	23/71 (32.4)	27/58 (46.6)	6/16 (37.5)	56/145 (38.6)
	2010	13/52 (25.0)	3/12 (25.0)	0/1 (0.0)	16/65 (24.6)
	2009	3/23 (13.0)	0/1 (100.0)	0/0 (0.0)	3/24 (12.5)
	2008	1/6 (16.7)	0/0 (0.0)	0/0 (0.0)	1/6 (16.7)

#### Range of Functions/Functionality

Examining the range of functions of diabetes apps demonstrated that most were limited to one function (54.1%, 355/656). Only 185/656 (28.2%) combined two functions, and three or more functions were offered by 116/656 (17.7%) of the apps available as of April 2013 (Table 5). Apps developed exclusively for iOS tended to offer a wider range of functions compared to Android apps.

**Figure 3:**
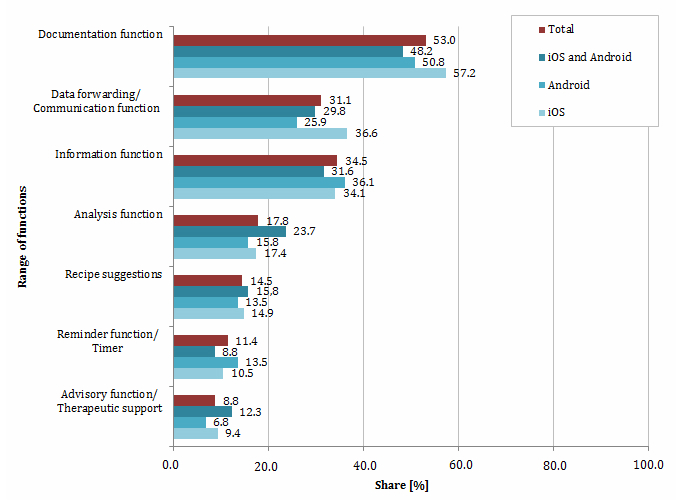
Range of functions of diabetes apps available as of April 2013.

A total of 348/656 (53.0%) apps and thus the majority of diabetes apps available as of April 2013 offered a documentation function (Figure 3). By using this feature, the measured blood glucose values can be recorded and either summarized in a table or plotted as a graph. Hence, the app enables the user to monitor the disease progression.

The recording of the blood glucose values mainly occurred via manual data input. Only a small number of apps offered the option to transfer the data wirelessly and automatically from the measuring device via Bluetooth to the mobile device.

The documentation function may be linked with an analysis function, which opens up the possibility to analyze the recorded data and to graphically display the results (Multimedia Appendix 2); 117/656 (17.8%) of the diabetes apps offered this service (Figure 3). The documentation function includes the recording and monitoring of individual eating habits (eg, the bread unit intake). Some apps, additionally, log the frequency of the user’s physical activity or the individual medical therapy (type and frequency). The documentation function was frequently linked with a reminder function, which reminds the user of its periodic, pre-defined medication (11.4%, 75/656) (Figure 3). According to the holistic setting approach, some of the available apps already offered the opportunity to track the course of disease for affected family members.

In total, 226 (34.5%) of the examined diabetes apps offered an information function, including the ability to inform about the illness, its diagnosis, the course of the disease, various treatment options, medication, and secondary diseases (Figure 3). Sometimes those apps provided information on the nutrient content of diverse foods and beverages and calorie consumption during various sporting activities (Multimedia Appendix 3).

A data forwarding/communication function was offered by 204/656 (31.1%) apps. With this function, the user has the opportunity to send the recorded data via email to the attending physician, family members, and/or friends (Multimedia Appendix 4). The reports can be sent frequently or on demand. Some of the apps were connected to special diabetes forums, where the users can upload their individual blood glucose values and discuss them with other diabetes patients (name: Diabesties, developer: Ayogo Health).

Surprisingly, only 58/656 (8.8%) of the diabetes apps provided an advisory function or any other kind of therapeutic support (Figure 3). Only a limited number of apps used the recorded data to create individualized advice to optimize the patients measuring, medication, eating habits, or activity behavior. One reason may be a previously required certification as a medical product for that kind of support.

Besides the previously described functions, 95/656 (14.5%) of the apps included suggestions for recipes suitable for the needs of diabetics (Figure 3).

As an example, Multimedia Appendix 5 shows screenshots of a highly reviewed app linking a documentation, analysis, communication, and information function (name: IBG Star Diabetes Manager mg/dl, developer: Sanofi Diabetes).

**Table 5:** Number of functions, target user groups, and popularity/user ratings of diabetes apps available as of April 2013.

Category	Subcategory	Operating system			
		iOS (n=276)	Android (n=266)	iOS and Android (n=114)	Total (n=656)
**Number of functions per diabetes app, n (%)**					
	1 function	134 (48.6)	156 (58.6)	65 (57.0)	355 (54.1)
	2 functions	87 (31.5)	71 (26.7)	27 (23.7)	185 (28.2)
	3 functions	36 (13.0)	25 (9.4)	13 (11.4)	74 (11.3)
	4 functions	15 (5.4)	11 (4.1)	9 (7.9)	35 (5.3)
	> 4 functions	4 (1.4)	3 (1.1)	0 (0.0)	7 (1.1)
**Target user groups, n (%)**					
	Patients	263 (95.3)	260 (97.7)	107 (93.9)	630 (96.0)
	Physicians/qualified health personnel	19 (6.9)	17 (6.4)	14 (12.3)	50 (7.6)
	Patients and physicians/qualified health personnel	6 (2.2)	11 (4.1)	7 (6.1)	24 (3.7)
**Popularity/user ratings**					
	Share of apps with rating, n (%)	31 (11.2)	189 (71.0)	75 (65.8)	295 (45.0)
	Median number of ratings	9.0	6.0	6.0	7.0
	Median number of stars (max 5)	3.5	4.0	4.0	3.8

#### Target User Groups

The vast majority (96.0%, 630/656) of the examined apps were designed specifically for patients, 24/656 (3.7%) apps addressed both patients and physicians/qualified health personnel, and only 50/656 (7.6%) were specifically designed for the target group physicians/qualified health personnel (Table 5). One reason might be the high potential for individual treatment and management of diabetes mellitus by the patients themselves. Particularly, patients suffering from type 2 diabetes have the opportunity to impact the course of the disease in a positive manner by a change in their lifestyles [31-33]. In this regard, diabetes mellitus differs from other chronic diseases such as cancer or dementia.

#### Popularity and User Rating

User ratings are a highly valuable and realistic evaluation of the additional benefits of apps. It is thus remarkable that just 31/656 (11.2%) of the apps designed exclusively for iOS were rated by users. In comparison, 189/266 (71.0%) of the Android apps and 75/114 (65.8%) of the apps running on both operating systems were rated (Table 5). One reason might be the rating procedure of the Apple App Store, which is more complicated than the procedure of the Google Play Store and requires several steps to rate an app. The median of the amount of provided ratings varied between six (iOS & iOS/Android apps) and nine ratings (Android apps). With a maximum of five stars for an app evaluation, the median rating varied from 3.5 (iOS apps) to 4 (Android & iOS/Android apps) stars (Table 5). Thus, 50% of the diabetes apps earned ratings of more than 3.5 to 4 stars, corresponding to a moderate to good rating.

Not only was the lower number of rated iOS apps conspicuous, the median of awarded stars was also lower than for Android apps (Table 5). That was surprising due to the higher access restrictions (peer review-based admission procedure for new apps) by the Apple App Store compared to non-existent restrictions by the Google Play Store.

Except for the ratings, the Google Play Store gave information about the number of downloads (ie, the number of installations) as another indicator of the app popularity. This information was not given by the Apple App Store. Hence, it was not possible to compare this indicator between both operating systems. But it has been shown that the number of downloads tended to correlate with the number of ratings and awarded stars.

#### Connection Between Acquisition Costs and User Ratings

**Figure 4:**
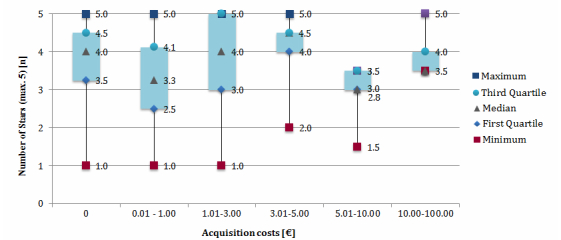
Distribution of user rating differentiated by acquisition costs as of April 2013.

During the analysis, the question arose of whether there is a connection between the price of an app and the level of user ratings. The results indicated that there existed a positive correlation between the acquisition costs and the number of given stars, for the price range of €0.01 to €5.00 (Figure 4). If the price exceeded €5.00, the correlation tended to inverse and the apps received worse evaluations. However, compared to free apps, no clear differences in the number of given stars could be found.

In general, free apps were rated more frequently than paid apps. With a share of 56.5% (204/361), they received the highest number of given ratings compared to just 27.5% (28/102; price range: €0.01-€1.00) up to 41.7% (5/10; price range: €10.00-€100.00) of the paid apps. However, it has to be considered that the number of free apps was considerably higher than the number of price-intensive apps.

#### Availability of Interfaces to External Sensors/Devices

Contrary to our initial expectations, only a limited number of diabetes apps possessed an interface to an external sensor or a measuring device (eg, for the measurement of blood glucose). Predominantly, apps developed for both operating systems were able to connect with an external sensor/device (7.9%, 9/114). Rarely, iOS apps (2.5%, 7/276) offered this feature compared to Android apps (5.3%, 14/266).

The majority of apps that were able to connect to an external measuring device transmitted the data via a Bluetooth interface. This interface enabled a wireless data transfer to the mobile device or to a PC. Some of the measuring devices already offered an automated transmission of the measured values in real time. There were two options for data synchronization: (1) wireless transfer of measured values to a mobile device and synchronization with the Internet, mostly to an online patient diary (registration required), and (2) wireless transfer of measured values to a PC, transfer of data to an online platform (registration required), and synchronization with a mobile device in the second step (eg, System Health Vault via Microsoft).

### Expert-Based Usability Evaluation

In total, we evaluated 66 out of 656 diabetes apps within the usability evaluation (Multimedia Appendix 6): 29 apps available exclusively for the operating system iOS, 28 apps available exclusively for the operating system Android, and 9 apps available for both operating systems.

For all main and subcriteria, we averaged the evaluations of all three experts. The values of the main criteria represent the mean of the corresponding subcriteria (Table 6). The total usability score was calculated from all categories, which were determined by means of a 5-point Likert scale.

Analyzing the results, the majority of evaluations were in the range of 3.0 to 4.0, which corresponded to a moderate to good rating of the apps included in the 10% sample. All tested apps received the best rating for the subcriteria “use of understandable semantics” and “simple comprehensibility and interpretability of displayed images and depictions” with a total average value of 4.1 (Table 6). Independent of the operating system, all apps received the worst rating for the subcriterion “fault tolerance” (2.8) followed by “simple recognizability of click-sensitive areas” (3.0). It has to be mentioned that it was only possible to evaluate fault tolerance for apps offering data input (36.4%, 24/66) (Table 6). The values determined for all other subcriteria varied between 3.1 and 4.0. Comparing the total usability score between the different operating systems, we found no clear differences with values varying between 3.3 and 3.4 for both iOS/Android apps. The worst-rated app with a usability score of 2.5 was HealthFile LIVE! (developer: WakefieldSoft LLC) for Android (Multimedia Appendix 6). The best-rated app was Diabetic Recipes Volume I (developer: ECI) for Android with a usability score of 4.1. Password-protected services were offered by an average of 18.2% of the apps.

In the second run, we evaluated the three chosen accessibility features. The results show that their operability was rather limited. The highest values were observable for the screen reader features Voice over (iOS) and Talkback (Android); 25 (86.2%) of the 29 iOS apps offered the ability to read the screen content aloud compared to 19 (67.9%) of the 28 Android apps, and just 4 (44.4%) of the 9 apps designed for both operating systems. The feature “invert colors” showed no considerable improvement of color contrast compared to the results of our evaluation without testing this feature. The results for testing the feature “large type” differed widely. While none of the iOS apps offered this feature, 11/27 (40.7%) Android apps offered contents in large font (Table 6).

While conducting our systematic review, we hypothesized that usability decreases with an increasing number of functions. Hence, we additionally investigated the relationship between the main usability criteria and the number of functions by conducting several correlation analyses. The results shown in Table 7 indicate statistically significant negative relationships between the number of functions and all usability criteria, except “fault tolerance”. Thus, the number of functions and all usability criteria were significantly negative correlated with coefficients varying between −.29 and −.25 implicating that diabetes apps offering a wider range of functions performed worse in terms of usability.

Furthermore, we analyzed the relationship between the usability score and specific functions based on the differences in functionality we found in our systematic review. Therefore, we conducted multiple linear regression analysis to control for potential confounding effects of other functions offered by the same app (Table 8). It showed significant results only for two types of functions. The presence of a documentation function reduced the usability score on average by 0.36 points while the usability score of apps offering an analysis function was on average reduced by 0.21 points. According to *R* ^2^, 25% of the variance of the usability score was explained by the model. All correlation and regression analyses were conducted with the statistical software Stata 11.1.

**Table 6:** Usability scores from expert-based usability evaluation by operating system, shown as mean values.

Main criterion	Subcriteria	Operating system			
		iOS (n=29)	Android (n=28)	iOS and Android (n=9)	Total (n=66)
		mean (SD)			
**Comprehensibility**		4.1 (0.53)	4.0 (0.43)	3.7 (0.35)	4.0 (0.48)
	Use of understandable semantics	4.3 (0.58)	4.0 (0.45)	3.8 (0.45)	4.1 (0.54)
	Simple comprehensibility and interpretability of displayed images and depictions	4.2 (0.54)	4.1 (0.53)	4.0 (0.37)	4.1 (0.51)
	Simple, self-explanatory menu structures	3.7 (0.82)	3.9 (0.84)	3.3 (0.66)	3.7 (0.82)
**Presentation (Image and Text)**		3.4 (0.36)	3.6 (0.38)	3.2 (0.36)	3.5 (0.40)
	Sufficient color contrast	3.5 (0.52)	3.8 (0.47)	3.1 (0.89)	3.6 (0.60)
	Sufficient color contrast with accessibility feature “invert colors”	3.2 (0.65)	3.9 (0.55)	3.4 (0.56)	3.5 (0.68)
	Big size of operating elements	3.4 (0.69)	3.2 (0.57)	3.1 (0.18)	3.3 (0.59)
	Ability to adapt the size of operating elements and displayed images^a^, n (%)	8 (27.6%)	4 (14.3%)	2 (22.2%)	14 (21.2%)
	Ability to adapt the size of operating elements and displayed images with accessibility feature “large type”^a^, n (%)	0 (0.0%)	11 (40.7%)^b^	3 (37.5%)^b^	14 (21.2%)
**Usability**		3.4 (0.43)	3.2 (0.44)	3.2 (0.38)	3.3 (0.43)
	Instant and easily understandable feedback	3.3 (0.66)	3.3 (0.53)	3.5 (0.47)	3.3 (0.58)
	Intuitive usability	3.6 (0.68)	3.5 (0.72)	3.3 (0.56)	3.5 (0.68)
	Simple recognition of click-sensitive areas	3.1 (0.65)	2.8 (0.45)	2.9 (0.48)	3.0 (0.55)
	Accessibility Features: Voice over (iOS), Talkback (Android)^a^, n (%)	25 (86.2%)	19 (67.9%)	4 (44.4%)	48 (72.7%)
**General characteristics**		2.5 (0.95)	2.8 (0.87)	3.5 (0.43)	2.8 (0.89)
	Fault tolerance/Efficient fault management	2.5 (0.95)	2.8 (0.87)	3.5 (0.43)	2.8 (0.89)
	Password-protected services^a^, n (%)	5 (17.2%)	4 (14.3%)	3 (33.3%)	12 (18.2%)
Number of functions per app		1.6 (0.82)	1.7 (0.85)	1.6 (1.13)	1.7 (0.89)
Total Usability Score		3.3 (0.40)	3.3 (0.38)	3.4 (0.48)	3.3 (0.39)

^a^The values of this subcriterion show means of frequencies.

^b^One observation was missing for this subcriterion and the corresponding operating system. Accordingly n is reduced by 1.

**Table 7:** Spearman’s rank correlation coefficients comparing number of functions with main usability criteria scores.

Number of functions	Main usability criteria scores			
	Comprehensibility	Presentation	Usability	Fault tolerance
1	−.29* (*P*=.02)	−.25* (*P*=.046)	−.25* (*P*=.04)	.46** (*P*<.001)

*5% significance level

**1% significance level

**Table 8:** Multiple regression analysis: relationship between usability score and functions.^a^

Variable	Coefficient (b)	95% CI	*t*	*P*
Information function	−.11	−0.29 to 0.07	−1.23	.22
Recipe suggestions	.06	−0.15 to 0.27	0.58	.56
Documentation function	−.36	−0.57 to −0.15	−3.43	.001^b^
Analysis function	−.21	−0.39 to −0.02	−2.23	.03^c^
Reminder function/timer	−.04	−0.42 to 0.33	−0.23	.82
Advisory function/therapeutic support	−.12	−0.38 to 0.14	−0.90	.37
Data forwarding/communication function	.04	−0.20 to 0.27	0.31	.76
Intercept	3.72	3.53 to 3.91	38.97	<.001^b^
	n=66	*F*_7,58_=3.46	*R* ^2^=.25	

^a^Ordinary Least Squares regression with robust standard errors

^b^1% significance level

^c^5% significance level

## Discussion

### Systematic Review

The systematic review showed that a large number of diabetes apps are available. Providers may be entering the market as a result of the rising number of patients suffering from diabetes. For users, especially patients, it becomes increasingly difficult to find an app in this plethora of options that is suitable for one’s own needs. This problem is caused by a lack of effective search criteria and filter functions in the app stores. More frequently, apps are chosen that appear first in the search results for diabetes apps. The sorting criteria in the app stores are not apparent. New apps from relatively unknown developers could have difficulties being listed among the first results.

At the same time, many apps offered similar functionalities, mostly a documentation function, which is consistent with earlier findings of Martínez-Pérez et al [3], Eng et al [5], Chomutare et al [6], and Demidowich et al [16]. Differences were found mostly in the design and the menu structure. Additionally, the majority of diabetes apps offered only one or two functions. An application that simultaneously informs and contributes to successful treatment by combining documentation, reminder, and advisory functions was not available as of April 2013. Such a multifunctional app would have a clear additional benefit, especially for newly diagnosed and elderly diabetes patients. At the same time, simple, understandable design, content, and menu navigation are needed. But several apps showed a lack of suitability and usability for its main target group diabetics, which is in accordance with the findings of Demidowich et al [16]. Some were apparently developed without intensive cooperation or prior (usability) tests with patients or professional health care personnel. The obligation for certification as a medical product does currently not exist, even though some diabetes apps are already certified, especially those linked to an external measurement device, eg, iBGStar Diabetes Manager (iOS), Bodytel Mobile (iOS and Android), or Diabetes Companion by mySugr (iOS and Android). Peer review processes of health apps are already offered by several platforms such as iMedical Apps [34], JMIR mHealth [35], or HealthOn [36]. This structure offers substantial and valuable support for users and their decision-making processes, but also in terms of quality assurance and improvement. In contrast, the Google Play Store, as one of the leading app stores, does not currently apply a peer review-based admission procedure for new apps. This lack of certification results in a lack of “[…] demonstrated safety and effectiveness, especially where information and trends are not just presented to patients, but used to make treatment recommendations” [37].

As an example, one app mainly offered labels like “after breakfast” or “after lunch” for the documentation of measured blood glucose values, which implies postprandial states. But, for most diabetics, the blood glucose values *before* eating are decisive to adjust the amount of insulin. Another app offered the feature to plot a graph labeled “HbA1c (glycated hemoglobin) according to day-time”. This neglects that the HbA1c is a value for long-term blood glucose. As a further example, some apps provided no option to modify, once entered, values at a later point in time. This misconception prevents a subsequent data amendment for example in the run-up of a doctors’ visit. At the same time, there is no opportunity to correct wrong values, for instance, as a result of erroneous data input.

Taking a look into the future, we expect that the data forwarding function, especially to the attending physician, will gain significantly more importance. A regular transmission of data to their physician linked with frequent feedback can be a valuable therapy support, particularly for people in rural regions that are or will become affected by a shortage of doctors [38,39]. Nevertheless, there still exist open questions concerning data security, network coverage, interoperability, documentation requirements, and coverage in health care plans, etc [38,40,41].

**Figure 5:**
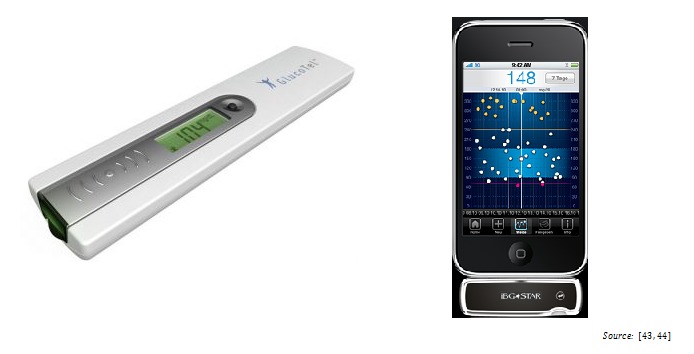
Glucose meters with automated transmission of blood glucose values to mobile devices.

Additionally, the automated transmission of measured values in real time from the measuring device to the mobile device will probably spread and is an important driver for the perceived ease of use as El-Gayar et al point out [42]. At the moment, the input of the measured blood glucose values occurs manually in most of the cases, as earlier findings of Eng et al [5] and Chomutare et al [6] confirm. Some manufacturers already offer blood glucose meters that allow real-time data transmission of measured blood glucose data via Bluetooth to a mobile device (eg, GlucoTel [43], iBGStar [44]) (Figure 5). This function simplifies the process of documenting for the patient and, at the same time, it increases the reliability of the entered data and subsequent analysis.

Notwithstanding the functions offered by diabetes apps, their effects on patients’ self-management and, accordingly, on important indicators, as for example the HbA1c value, have to be evaluated. A comprehensive, representative, and long-term study investigating these health effects is lacking so far. But different studies focusing on the outcomes of mobile phone interventions, such as SMS, point out a slightly positive influence as shown in the reviews of Holtz et al [45] and Free et al [46].

### Expert-Based Usability Evaluation

As a supplement to our systematic review, we conducted an expert-based usability evaluation to examine the usability of currently available diabetes apps for patients aged 50 or older. Therefore, we focused on the age group with the highest diabetes prevalence. The results show moderate to good evaluations (range 3.0-4.0) for all reviewed usability criteria, which is in accordance with the results of Demidovich et al [16]. An exception was “fault tolerance” (Table 6). The main criteria, “comprehensibility”, rated best with a score of 4.0. In particular, the elderly benefit from easy, understandable semantics and easy, comprehensible, and interpretable images and depictions, due to their frequently limited experience in handling mobile devices and apps. Hence, it can lower inhibition thresholds, especially during the first time of use, and therefore increase acceptance among this age group. The same is true for the influence of “easily understandable feedback” (3.3) and an “intuitive usability” (3.5) (main criterion “usability”). However, these two subcriteria performed worse within our evaluation. The fact that most of the diabetes apps were in English or contained English/foreign language terms (Table 3) diminished the usability especially for non-English-speaking elderly in terms of comprehensibility. This can be seen as one optimization approach for future app development that is comparatively easy to implement.

The evaluation showed moderate results for the main criterion “presentation” (3.5). Our test of three accessibility features indicated a very good operability of the screen readers, especially for Voice over (86.2%) offered by iOS. However, the operability of the features “invert colors” (3.5) and “large type” (21.2%) was rather restricted. Additionally, the minority of diabetes apps (17.8% of the iOS apps) were developed specifically for tablet PCs. However, we assess them as more suitable and user-friendly for elderly diabetes patients due to their larger display and bigger illustrations. With increasing age, cognitive and physical skills are declining, such as eyesight, visual acuity, color vision, contrast detection, and hearing [11]. Especially elderly diabetes patients are often suffering from retinopathy. Shortcomings of diabetes apps concerning the presentation of information (color contrast, size of operating elements, option to flexibly adapt size of operating elements, etc) and the operability of accessibility features are deterring potentially interested patients from using diabetes apps from the outset. Therefore, a barrier-free access is a basic prerequisite for elderly patients to make them use diabetes apps. Against this background, all the other criteria we determined become of secondary importance in terms of usability.

The criterion “fault tolerance” rated worst with a score of 2.8 (Table 6). This means that the available diabetes apps were lacking an efficient fault management (criterion specified in Table 2). Especially inexperienced (elderly) users often have difficulties with inputting data. Some errors are unrecoverable or even cause the application to shut down, as Garcia et al already demonstrated in their analysis [17]. These results have to be viewed with great concern due to the fact that these apps are dealing with medical parameters. This becomes particularly serious if these values provide the basis for further calculations as, for example, the required dose of insulin. Our results demonstrate once again the meaningfulness of an automated transfer of measured values from the blood glucose meter to the mobile device [42]. Additionally, it could be helpful to limit choice to meaningful values, for example, by offering a numeric keyboard to enter blood glucose values.

Our correlation and regression analyses indicated a strong link between usability and the number and kind of functions. In particular, the number of functions and all main usability criteria were significantly negative correlated. These results cast a different light on the aforementioned outcomes of our usability test. Hence, the moderate to good usability scores applied mainly to apps offering a small range of functions. This relation inverted when we looked upon the considerably lower usability scores for multifunctional apps (Multimedia Appendix 6). Considering the special needs of elderly diabetes patients, they would benefit from a comprehensive and easily understandable support as already mentioned above. They are frequently affected by multimorbidity and polypharmacy, particularly after many years of suffering from diabetes [47].

Differed by functions, apps offering a documentation and an analysis function performed worse in terms of usability. This result is surprising as the documentation function is most commonly offered with a share of 53.0%. It can be a valuable support for all diabetes patients measuring and recording blood glucose level regularly. But as interviews with diabetes patients aged 50 or older (conducted in Germany in 2013) have shown, most of them prefer documentation by means of a conventional diary (results not shown). One reason they named was the aforementioned lack of usability and therefore a too complicated and time-consuming handling. Moreover, the use of these two functions is characterized by a higher level of human-technology interaction than, for example, the use of an information function. Of course, this can be accompanied by a wider scope of error sources and usability barriers.

Altogether, the potential of diabetes apps for assisting and supporting diabetes patients aged 50 or older is large. In particular, the target group aged between 50 and 60 years holds great potential as people of this age are already quite familiar with mobile devices and apps [48]. Now, app developers are facing the challenge of taking sufficient account of the usability criteria we examined and addressing those shortcomings. There is no need for a huge number of new app functions. It is more about improving what already exists.

### Future Work

The systematic review and the expert-based usability test were conducted within the project “InnoMedTec”. In that project, we investigate the question: “How should a mobile application be designed to support an effective self-management for diabetes patients aged 50 or older?” Our market analysis provided the basis for a survey among diabetes patients aged 50 or older and physicians, which we conducted in the second half of 2013. Within guided interviews, we investigated the current use, acceptance promoting/inhibiting factors, potentially needed support, and concrete design features for the development of a diabetes app. Merging the results of the systematic market review and the survey, a user- and needs-oriented prototype app for diabetics aged 50 or older will be developed this year. To guarantee usability and needs orientation, the prospective users and usability experts are involved in the product development process right from the beginning. User- and expert-based usability tests are performed regularly. The results are integrated continuously in the app optimization until its finalization.

### Limitations

#### Systematic Review

The conducted review focused exclusively on apps for the currently leading operating systems, iOS and Android. Currently available diabetes apps for other operating systems, such as Windows Phone, Blackberry OS, or Symbian, were not considered within the analysis. The app publication date was solely available for iOS apps, but not for Android apps. Here, the date of the last update served as reference value. Due to that fact, the results concerning the annually new released diabetes apps were not directly comparable.

The app information was gathered by studying the descriptions in the app stores and within the app itself. More detailed information, such as download statistics, were not available for analysis. Perhaps this information would enable more detailed results concerning the user groups, for example, differentiated by gender, age groups, or type of diabetes.

#### Expert-Based Usability Evaluation

Within our usability evaluation, we investigated usability criteria exclusively. We evaluated neither the quality of content and functions nor their effectiveness. Furthermore, it has to be mentioned that one usability evaluation cannot claim to cover all possible and critical usage situations that can possibly occur [18-20,49].

We would also stress that we examined a sample of all available diabetes apps, not just a sample of apps developed specifically for the elderly. Hence, many of the apps we evaluated do not claim to be particularly suitable for this age group.

### Conclusions

Despite the huge amount of currently available diabetes apps, most of them offer a small number of similar functionalities. Patients and physicians should be directly involved during the app development to tackle the lack of usability and needs-orientation for its main target group diabetics. We think that data forwarding options and automated transmission of measured values to mobile devices will gain more importance in the future.

The usability of diabetes apps for patients aged 50 or older was moderate to good. But this result applied mainly to apps offering a small range of functions. Multifunctional apps performed considerably worse in terms of usability. Differed by functions, the documentation and analysis function indicated significantly lower usability scores. The operability of accessibility features for diabetes apps was quite limited, except for the feature “screen reader”.

## Multimedia Appendix 1

Dataset diabetes app review.

## Multimedia Appendix 2

Screenshots of apps with documentation and analysis function.

## Multimedia Appendix 3

Screenshots of apps with information function.

## Multimedia Appendix 4

Screenshots of apps with communication function.

## Multimedia Appendix 5

Screenshots of a highly reviewed app with various functionalities.

## Multimedia Appendix 6

Usability scores differed by operating systems and apps.

## Abbreviations

Apps: mobile applications
HbA1c: glycated hemoglobin

## References

[1] Ingraham N (2013). Apple: 900,000 apps in the App Store, 375,000 iPad-optimized, 28 million copies of mountain lion sold.

[2] MHealth Watch (2013). Mobile health care apps growing fast in number.

[3] Martínez-Pérez B, de la Torre-Díez I, López-Coronado M (2013). Mobile health applications for the most prevalent conditions by the World Health Organization: review and analysis. J Med Internet Res.

[4] Cafazzo JA, Casselman M, Hamming N, Katzman DK, Palmert MR (2012). Design of an mHealth app for the self-management of adolescent type 1 diabetes: a pilot study. J Med Internet Res.

[5] Eng DS, Lee JM (2013). The promise and peril of mobile health applications for diabetes and endocrinology. Pediatr Diabetes.

[6] Chomutare T, Fernandez-Luque L, Arsand E, Hartvigsen G (2011). Features of mobile diabetes applications: review of the literature and analysis of current applications compared against evidence-based guidelines. J Med Internet Res.

[7] Lehnert H, Wittchen HU, Pittrow D, Bramlage P, Kirch W, Böhler S, Höfler M, Ritz E (2005). Prevalence and pharmacotherapy of diabetes mellitus in primary care. Dtsch Med Wochenschr.

[8] Kirch W (2008). Encyclopedia of Public Health: Volume 1: A - H Volume 2: I - Z.

[9] Du Y, Heidemann C, Scheidt-Nave C, Robert-Koch-Institut (2011). Diabetes mellitus in Deutschland.

[10] International Diabetes Federation (2013). Diabetes Atlas.

[11] Holzinger A, Searle G, Nischelwitzer A, Stephanidis C (2007). On some aspects of improving mobile applications for the elderly. UAHCI'07 Proceedings of the 4th International Conference on Universal Acess in Human Computer Interaction. Coping with Diversity.

[12] Lorenz A, Oppermann R, Zahl L, Mielke D, Cheok AD, Chittaro L (2007). Personalized mobile health monitoring for elderly. MobileHCI ’07 Proceedings of the 9th International Conference on Human Computer Interaction with Mobile Devices and Services.

[13] Mallenius S, Rossi M, Tuunainen VK (2010). Factors affecting the adoption and use of mobile devices and services by elderly people – results from a pilot study.

[14] Lorenz A, Oppermann R (2009). Mobile health monitoring for the elderly: Designing for diversity. Pervasive and Mobile Computing.

[15] Steele R, Lo A, Secombe C, Wong YK (2009). Elderly persons' perception and acceptance of using wireless sensor networks to assist healthcare. Int J Med Inform.

[16] Demidowich AP, Lu K, Tamler R, Bloomgarden Z (2012). An evaluation of diabetes self-management applications for Android smartphones. J Telemed Telecare.

[17] Garcia E, Martin C, Garcia A, Harrison R, Flood D, Holzinger A, Simonic KM (2011). Systematic analysis of mobile diabetes management applications on different platforms. Information Quality in e-Health. Lecture Notes in Computer Science.

[18] Sarodnick F, Brau H (2011). Methoden der Usability Evaluation: Wissenschaftliche Grundlagen und praktische Anwendungen. 2nd ed.

[19] Nielsen J (1993). Usability engineering.

[20] Barnum CM (2011). Usability testing essentials: ready, set...test!.

[21] Gondy L (2011). Designing user studies in informatics.

[22] Abascal J, Arrue M, Fajardo I, Garay N, Navarro-Prieto R, Vidal JL (2006). An expert-based usability evaluation of the EvalAccess web service. HCI related papers of Interacción 2004.

[23] Lee YS (2007). Older adults’ user experience with mobile phones: identification of user clusters and user requirements.

[24] Deutscher Bundestag (2010). Sechster Bericht zur Lage der älteren Generation in der Bundesrepublik Deutschland - Altersbilder in der Gesellschaft.

[25] Caprani N, O'Connor NE, Gurrin C (2012). Touch screens for the older user.

[26] DIN-Fachbericht 131:2003 (2003). Leitlinien für Normungsgremien zur Berücksichtigung der Bedürfnisse von älteren Menschen und von Menschen mit Behinderungen; Deutsche und englische Fassung des CEN/CENELEC-Leitfadens 6.

[27] Schmid A, Dörfler I, Dany F, Böpple O, Shire KA, Leimeister JM (2012). Analyse der Akzeptanzkriterien für mobile Anwendungen im Bereich Gesundheit in der Zielgruppe 50+. Technologiegestützte Dienstleistungsinnovation der Gesundheitswirtschaft.

[28] Renaud K, van Biljon J, Navarro-Prieto R, Vidal JL (2008). Predicting technology acceptance and adoption by the elderly : a qualitative study. SAICSIT '08 Proceedings of the 2008 Annual Research Conference of the South African Institute of Computer Scientists and Information Technologists on IT Research in Developing Countries: Riding the Wave of Technology.

[29] Banse M (2008). Softwareergonomische Optimierung Touchscreen basierter Mensch-Computer-Interaktion.

[30] DIN EN ISO 9241-110:2008-09 (2008). Ergonomie der Mensch-System-Interaktion - Teil 110: Grundsätze der Dialoggestaltung. Deutsche Fassung EN ISO 9241-110:2006.

[31] Tuomilehto J, Lindström J, Eriksson JG, Valle TT, Hämäläinen H, Ilanne-Parikka P, Keinänen-Kiukaanniemi S, Laakso M, Louheranta A, Rastas M, Salminen V, Uusitupa M, Finnish Diabetes Prevention Study Group (2001). Prevention of type 2 diabetes mellitus by changes in lifestyle among subjects with impaired glucose tolerance. N Engl J Med.

[32] Diabetes Prevention Program (DPP) Research Group (2002). The Diabetes Prevention Program (DPP): description of lifestyle intervention. Diabetes Care.

[33] Knowler WC, Fowler SE, Hamman RF, Christophi CA, Hoffman HJ, Brenneman AT, Brown-Friday JO, Goldberg R, Venditti E, Nathan DM, Diabetes Prevention Program (DPP) Research Group (2009). 10-year follow-up of diabetes incidence and weight loss in the Diabetes Prevention Program Outcomes Study. Lancet.

[34] (2013). iMedical Apps.

[35] (2013). JMIR mHealth.

[36] (2013). HealthOn.

[37] Klonoff DC (2013). The current status of mHealth for diabetes: will it be the next big thing?. J Diabetes Sci Technol.

[38] Nuffield Council on Bioethics (2010). Medical profiling and online medicine: the ethics of 'personalised healthcare' in a consumer age.

[39] Ascher M (2011). Telemedicine in diabetes care.

[40] VDE Initiative Mikromedizin (2008). VDE-Positionspapier Telemonitoring zur Prävention von Diabetes-Erkrankungen.

[41] World Health Organization (2010). Telemedicine: opportunities and developments in member states: report on the Second Global Survey on eHealth 2009.

[42] El-Gayar O, Timsina P, Nawar N, Eid W (2013). Mobile applications for diabetes self-management: status and potential. J Diabetes Sci Technol.

[43] BodyTel Europe GmbH (2013). Glucotel.

[44] Sanofi Diabetes (2013). iBGStar.

[45] Holtz B, Lauckner C (2012). Diabetes management via mobile phones: a systematic review. Telemed J E Health.

[46] Free C, Phillips G, Felix L, Galli L, Patel V, Edwards P (2010). The effectiveness of M-health technologies for improving health and health services: a systematic review protocol. BMC Res Notes.

[47] Wittchen HU, Group DS (2005). Diabetes in Deutschland. DETECT: Eine bundesweite Studie in 3.500 Hausarztpraxen und 55.000 Patienten.

[48] Online Publishers Association (2012). A portrait of today’s smartphone user.

[49] Nielsen J, Plaisant C (1994). Usability inspection. CHI ’94 Conference Companion, Human Factors in Computing Systems, Celebrating Interdependence.

