# Supplementary figures and images for: Mobile Applications for Diabetics: A Systematic Review and Expert-Based Usability Evaluation Considering the Special Requirements of Diabetes Patients Age 50 Years or Older
Source: J Med Internet Res. 2014 Apr 9;16(4):e104. doi: 10.2196/jmir.2968 (PMC4004144; doi:10.2196/jmir.2968)

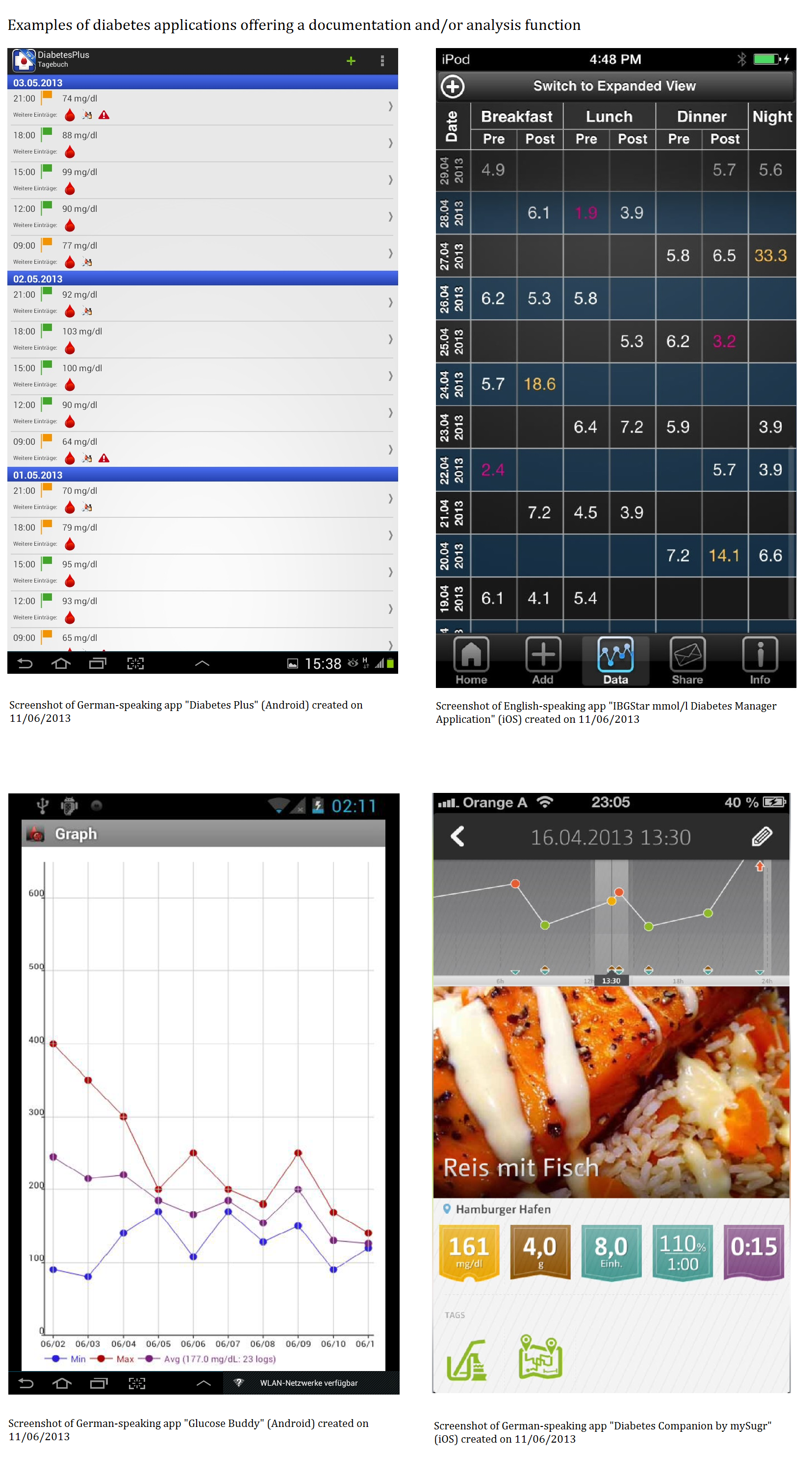

Supplement: Supplementary file 2 [file jmir_v16i4e104_app2.png]

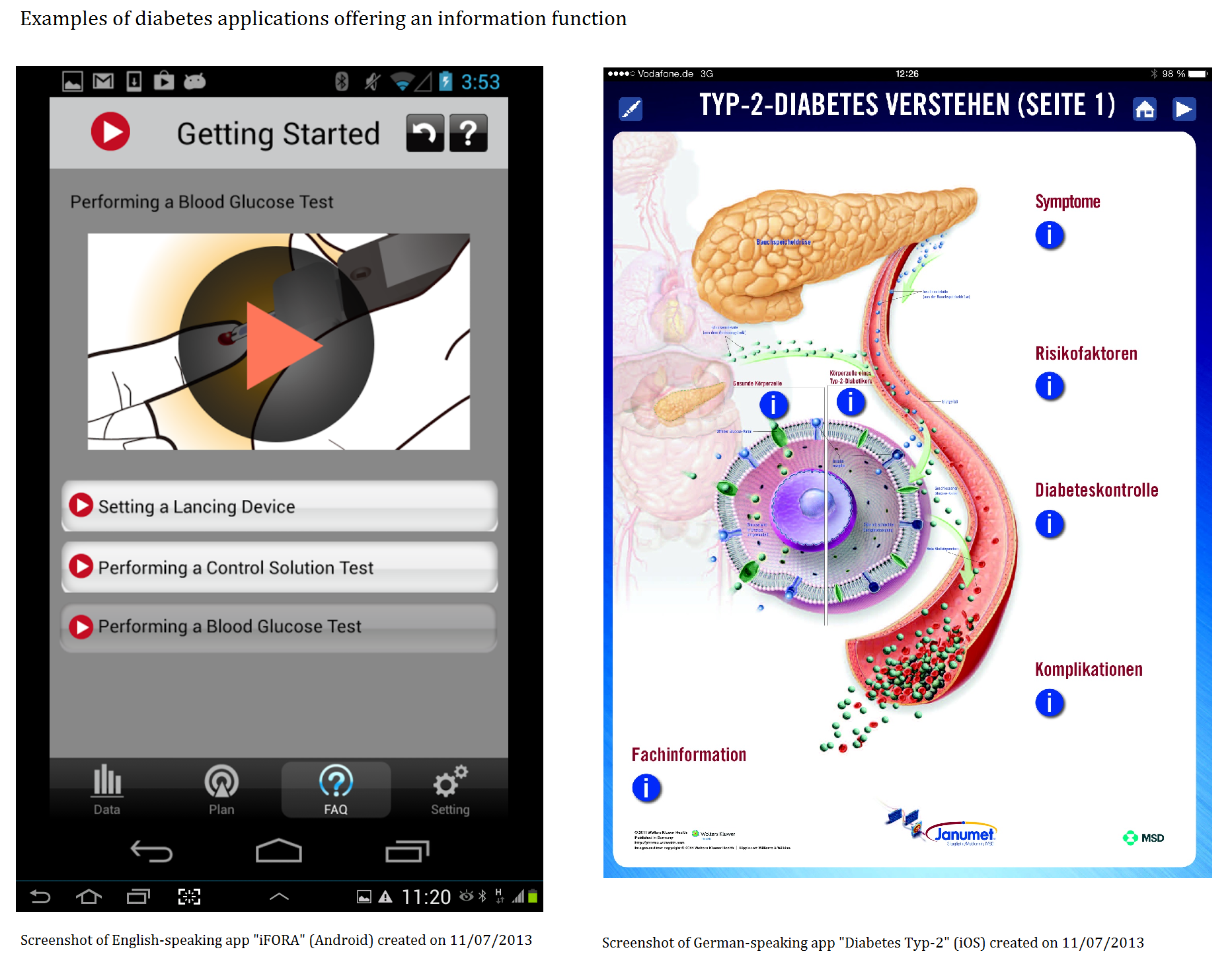

Supplement: Supplementary file 3 [file jmir_v16i4e104_app3.png]

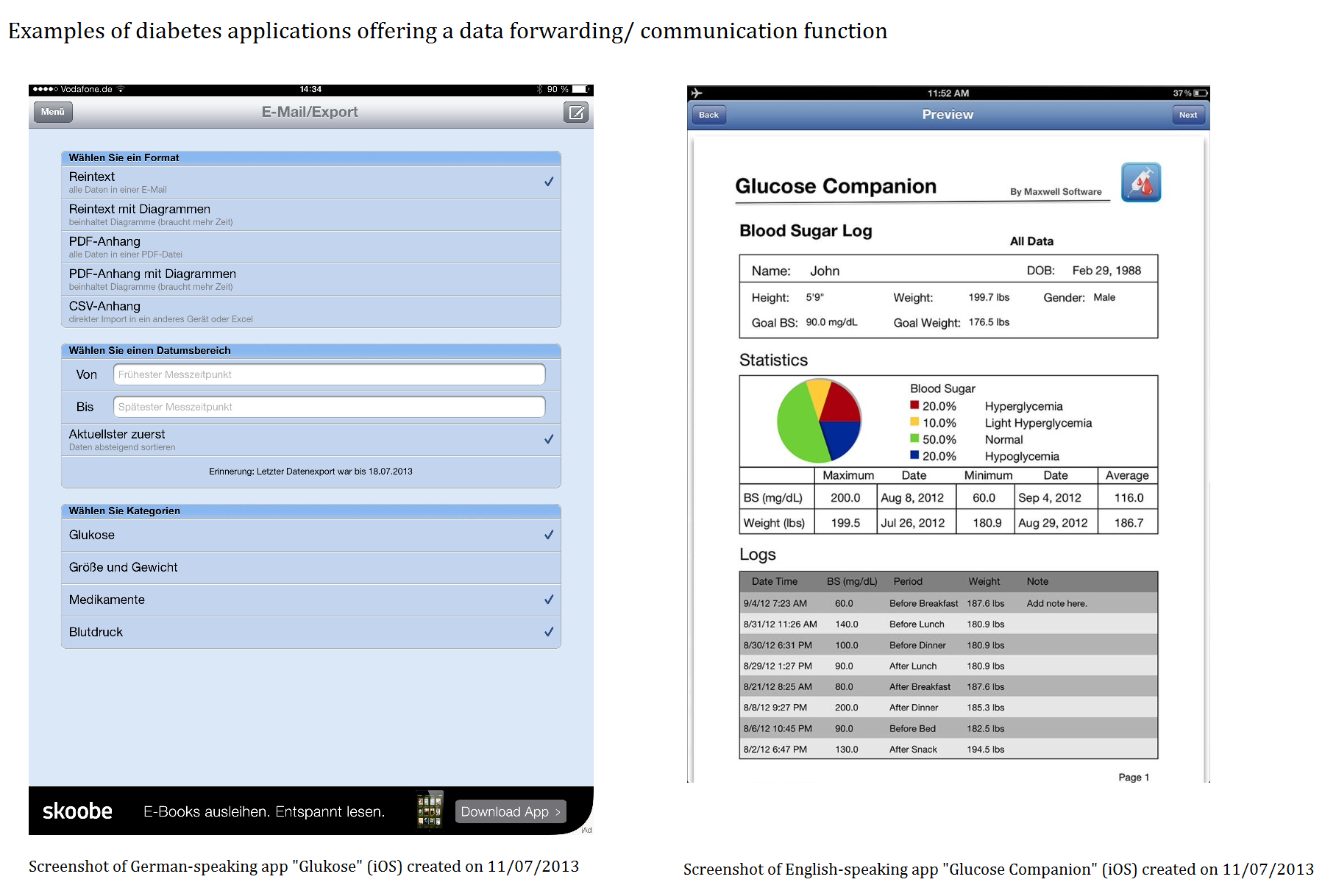

Supplement: Supplementary file 4 [file jmir_v16i4e104_app4.png]

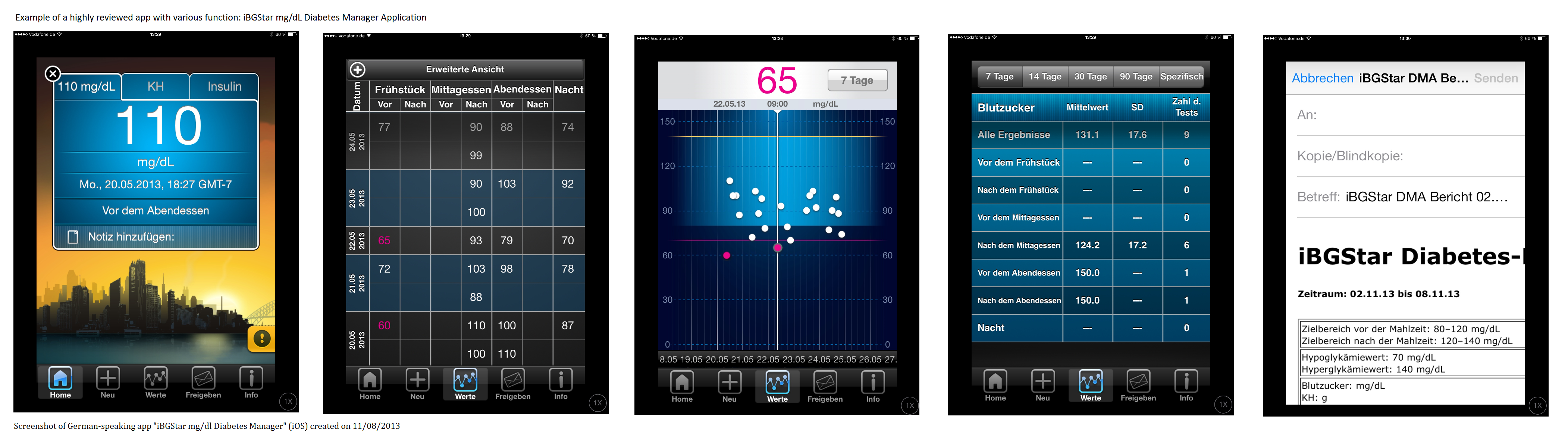

Supplement: Supplementary file 5 [file jmir_v16i4e104_app5.png]
